# Swimming of peritrichous bacteria is enabled by an elastohydrodynamic instability

**DOI:** 10.1038/s41598-018-28319-8

**Published:** 2018-07-16

**Authors:** Emily E. Riley, Debasish Das, Eric Lauga

**Affiliations:** 1 Department of Applied Mathematics and Theoretical Physics, University of Cambridge, Cambridge, UK; 2 Present Address: Centre for Ocean Life, Technical University of Denmark, Kongens Lyngby, DK-2800 Denmark

## Abstract

Peritrichously-flagellated bacteria, such as *Escherichia coli*, self-propel in fluids by using specialised motors to rotate multiple helical filaments. The rotation of each motor is transmitted to a short flexible segment called the hook which in turn transmits it to a flagellar filament, enabling swimming of the whole cell. Since multiple motors are spatially distributed on the body of the organism, one would expect the propulsive forces from the filaments to push against each other leading to negligible swimming. We use a combination of computations and theory to show that the swimming of peritrichous bacteria is enabled by an elastohydrodynamic bending instability occurring for hooks more flexible than a critical threshold. Using past measurements of hook bending stiffness, we demonstrate how real bacteria are safely on the side of the instability that promotes systematic swimming.

## Introduction

Although out of sight, bacteria dominate chemical processes on our planet. They are the most abundant organisms on earth and, equipped with the ability to live in extreme and hostile conditions, they play crucial roles in both the environment and human health^1^. Many bacteria self-propel in response to physical and chemical cues by actuating specialised, rotary motors in bulk fluid environments^2^. Each motor imposes a moment normal to the surface of the cell body transmitted to a helical flagellar filament via a short elastic segment called the hook that acts as a universal joint^3,4^. Due to the helical nature of flagellar filaments, the rotation imposed by each motor is not time-reversible and as a result bacteria are able to swim^5^. While a flagellar filament can take one of eleven polymorphic forms, the normal form used for swimming is left-handed and rotates counter-clockwise (CCW, looking from the flagellum to the cell) propelling the bacterium cell-first^6,7^, a type of swimmer known as a pusher^8^. If the same left-handed helix were to rotate in the opposite direction then the cell would swim flagella first and be a puller^9^.

**Figure 1:**
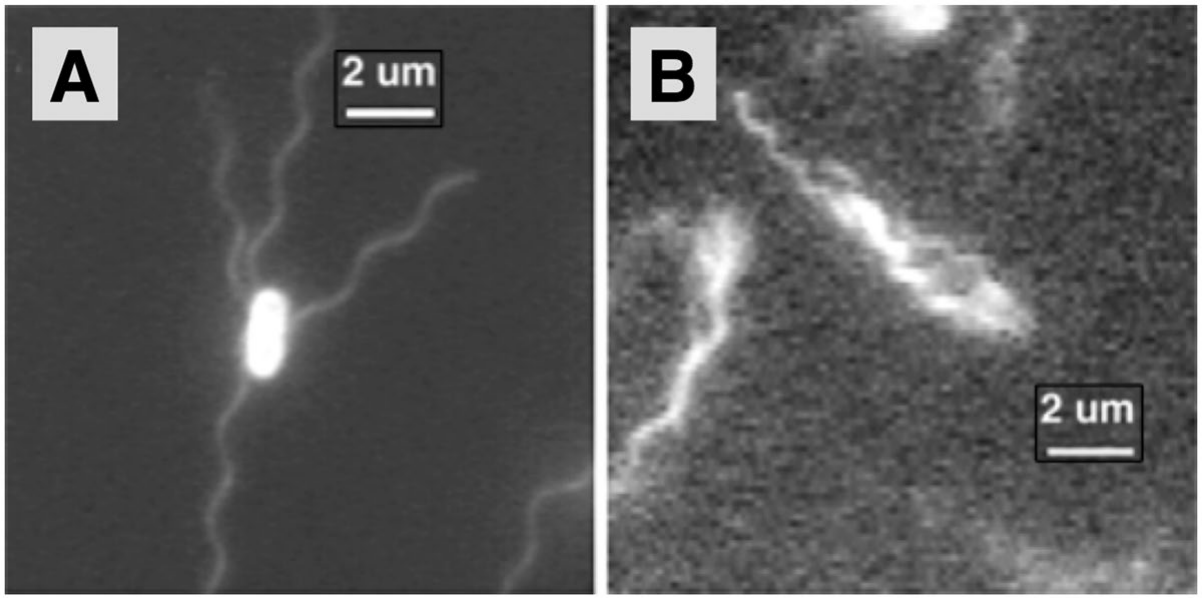
Swimming *E. coli* bacteria. (**A**) Peritrichous bacterium with multiple flagella spatially distributed around the cell body. (**B**) Flagellar filaments are located behind the swimming cell in a helical bundle whose rotation pushes the cell forward. Reproduced from Turner, Ryu & Berg (2000) *J. Bacteriol*., **182**, 2793–2801^6^. Copyright 2000 American Society for Microbiology.

Peritrichous bacteria possess multiple flagella that can grow from essentially any point on the cell body surface^10,11^. Well-studied examples include *Escherichia coli* (*E. coli*, Fig. 1A), *Bacillus subtilis* and *Salmonella enterica*. During the swimming of these pusher cells, all flagellar filaments gather and bundle at one end of the body propelling the cell forward (Fig. 1B). The main advantage of possessing multiple flagella is not increased propulsion^12^ but rather the ability to change direction via tumbling. This occurs when at least one of the rotary motors slows down^13^ or reverses its direction^7^ causing the bundle to break-up (unbundling). At the end of a tumble, the motors return to their normal CCW state, the bundle reforms and the bacterium swims in a new direction. Through a modulation of the tumbling frequency, bacteria can move towards favourable environments^14^. Crucial for the formation of the filament bundle, and successful swimming, is the flexible hook. When the hook is stiffened, bacteria are stuck in a tumble mode and can barely swim^15^. Hook flexibility is also crucial for singly flagellated bacteria, enabling changes in swimming directions via buckling^16^ but causing unstable locomotion if it is too flexible^17^, an effect itself stabilised by the presence of multiple flagella (Frank, T. M. N., Michael D. G., Submitted, 2018).

Much theoretical work has been devoted to predicting the propulsion mechanisms of self-propelled bacteria^18^. Most studies assume a fixed relative position between helical filaments (or bundles) and the cell body, and modelling tools have been developed to address both swimming^8,19–21^ and the bundling/unbundling process^22,23^.

If peritrichous bacteria have similar filaments distributed spatially around their cell body, why are the flagella not all pushing against each other leading to negligible swimming? In this paper we use a combination of computations and theory to show that swimming is enabled by an elastohydrodynamic instability of the hook. If hooks are too rigid, flagellar filaments always point normal to the cell body surface and never bundle. In contrast, when the bending rigidity of the hook is below a critical threshold, the feedback between the flow induced by the flagella and hook bending leads to a conformational instability resulting in all the flagellar filaments gathered at the back of the cell, and net locomotion. This sharp transition from negligible to successful swimming is observed numerically with decreasing hook stiffness and we show that this instability can be rationalised using a simple model of a cell propelled by two straight active filaments. By examining past measurements of hook flexibility, we demonstrate that bacteria are safely on the swimming side of the instability.

## Results

### Modelling of multi-flagellated bacteria

**Figure 2:**
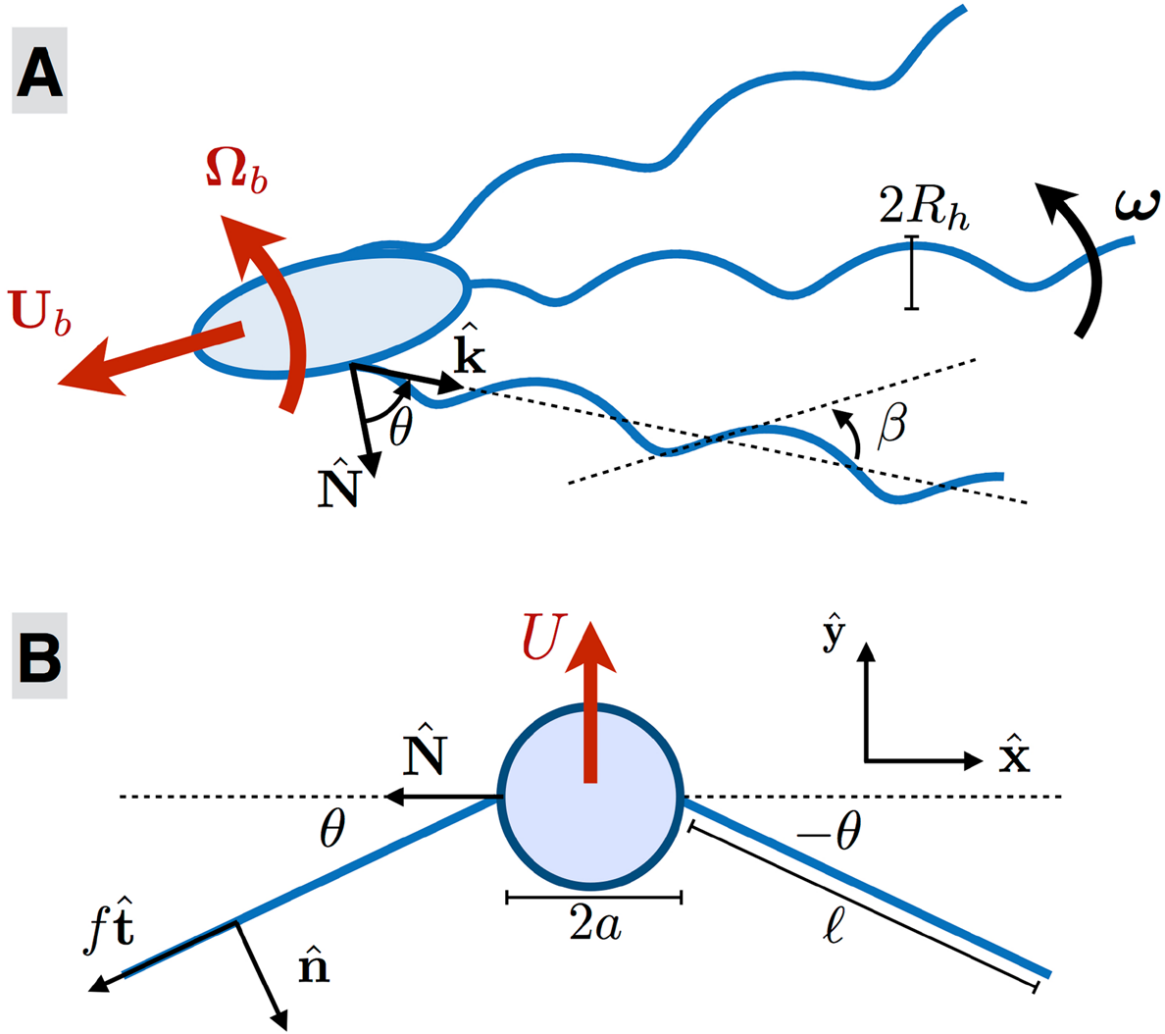
(**A**) Computational model of a peritrichous bacterium actuating *N*_*f*_ helical filaments (radius *R*_*h*_; pitch angle *β*) by rotating them about their axis $\hat{{\bf{k}}}$ with prescribed angular velocity. The flexible hook acts elastically to align the helix axis with the normal $\hat{{\bf{N}}}$ to the cell body. (**B**) Simplified model to capture the elastohydrodynamic instability. Two straight active filaments of length $\ell$ attached on either side of a spherical body of radius $a$ are tilted at an angle ±*θ* away from the cell body surface normal, $\hat{{\bf{N}}}$, and act on the cell with tangential force $f\ell \hat{{\bf{t}}}$ resulting in swimming of the model bacterium with velocity $U\hat{{\bf{y}}}$.

We start by building a computational model of the locomotion of a peritrichous bacterium, as outlined in the Methods section with mathematical details in supplementary information. We consider a bacterium propelled by *N*_*f*_ flagella (Fig. 2A) with a cell body in the shape of a prolate ellipsoid. Each flagellum consists of: (i) a rotary motor that generates a fixed rotation rate about the axis of the flagellar filament; (ii) a short flexible hook treated as a torsion spring about the motor axis whose hydrodynamics can be neglected^17^; (iii) a helical flagellar filament of the normal left-handed polymeric form whose hydrodynamics is captured with slender-body theory^24^. Motor and filament parameters are chosen to match those of *E. coli* bacteria^7^ (Table S1 in supplementary information). Each helical filament has a tapered end such that the helix radius is zero at its attachment point to the motor^25^. Flagellar filaments can rotate but not translate relative to their attachment point on the cell body and while the rotation about the helix axis is imposed by the motor, any further rotations relative to the body are solved for. We neglect hydrodynamic interactions between the cell body and flagellar filaments but include steric interactions to prevent filaments from entering the body. For each hook, we use *θ* to denote the tilt angle between the normal to the cell body at the motor location and the axis of the flagellar filament (i.e. when *θ* = 0 the filament is normal to the cell body). The restoring elastic moment imposed by each motor on its flagellar filament is modelled as torsion spring of spring constant $K=EI/{\ell }_{h}$, where *EI* and ${\ell }_{h}$ are the bending rigidity and length of the hook respectively^16^. The magnitude of the restoring moment is thus given by *K*|*θ*| and the elasticity of the hook acts to align the helix axis with the normal to the cell body. The computational model solves for the instantaneous positions of the flagellar filaments and for the swimming velocity, **U**_*b*_, and angular velocity, **Ω**_*b*_, of the cell body as a function of the hook stiffness.

### Pusher bacteria with flexible hooks undergo a swimming instability

**Figure 3:**
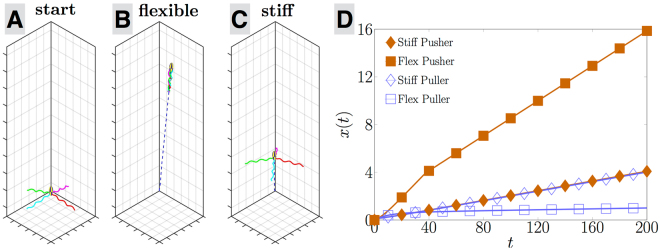
Swimming motion for a bacterium swimming with *N*_*f*_ = 4 flagella with a flexible vs. stiff hook. (**A**) Initial position and conformation of each cell. (**B**) Pusher cell with flexible hook at *t* = 200 (times scaled by rotation rate of flagella). (**C**) Pusher cell with stiff hook at *t* = 200. (**D**) Distance travelled by each swimmer (nondimensionalised by the pitch of the helical filaments) as a function of time for four different swimmers: stiff (diamonds) vs. flexible hook (squares) and pusher (filled symbols) vs. puller (empty).

Examining the results of our computational model uncovers a remarkable elastohydrodynamic instability, illustrated in Fig. 3 in the case of *N*_*f*_ = 4 flagella, the average number of flagella on an *E. coli* cell^6^. The motors are positioned symmetrically around the surface of the cell body. We start the computations with each flagellar filament tilted at some small angle away from the normal to the surface and march the system forward in time while tracking the position of the cell in the laboratory frame and of the flagellar filaments relative to the cell body. Associated movies are available online (see supplementary information).

In Fig. 3A–C we illustrate the trajectory of a pusher bacterium (i.e. a cell with flagellar filaments undergoing normal CCW rotation) with two different hook stiffnesses over a time scale *t* = 200 (time nondimensionalised by the rotation rate of the flagella). While both start at the same location (A), the cell with the flexible hook (*K* = 0.1) ends up with their flagellar filaments all wrapped in the back and is able to swim five times as fast (B) as the stiff-hooked cell (*K* = 100) whose flagellar filaments have remained in the same splayed configuration (C). This is quantified in Fig. 3D where we plot the net distance travelled as a function of time (scaled by the pitch of the helix). The cell with a flexible hook (filled square) swims consistently faster than the stiff one (filled diamond). If alternatively we reverse the direction of rotation of the flagella to rotate in the clockwise (CW) direction, the cell becomes a puller and does not transition to fast swimming for neither a flexible hook (empty squares) nor a stiff one (empty diamonds). Note that the two stiff cases (pushers and pullers; diamonds) have identical swimming magnitude, a consequence of the kinematics reversibility of Stokes flows^5^. Importantly, the transition to fast swimming for flexible pusher bacteria does not occur smoothly with changes in the hook stiffness but instead it takes place at a critical dimensionless value of *K*_*c*_ ≈ 1 (nondimensionalised using the viscosity of the fluid, the pitch of the helical filament and the frequency of rotation). Above *K*_*c*_, all flagella remain normal to the cell (*θ* ≈ 0) leading to negligible swimming while below *K*_*c*_, all flagella wrap behind the cell (|*θ*| ≈ *π*/2) leading to a net locomotion.

**Figure 4:**
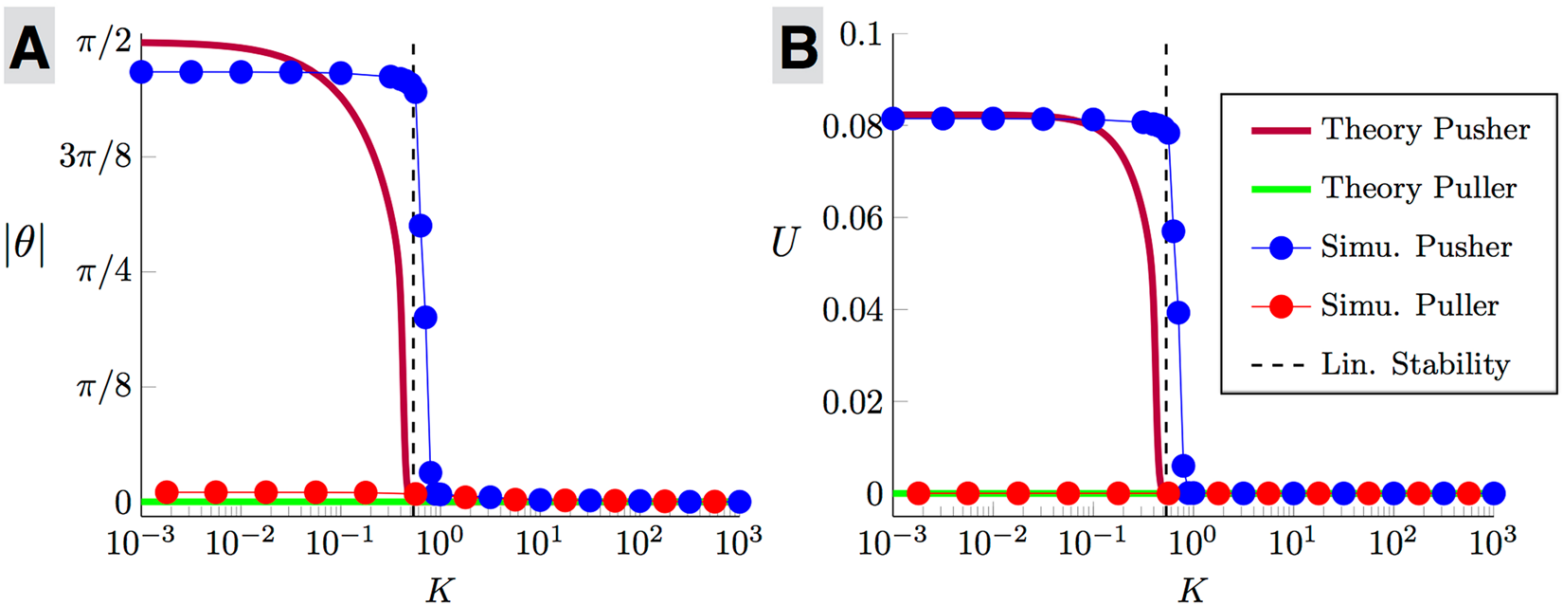
Steady-state flagella tilt angles (|*θ*|, **A**) and lab-frame swimming speeds (*U*, **B**) for the full computational model of Fig. 2A with two flagella (symbols and thin lines) and for the simple active filament model of Fig. 2B (thick lines) as a function of the dimensionless hook spring constant, *K*. Green line and light red symbols: puller bacterium for which the non-swimming state is always stable; Dark red line and dark blue symbols: pusher bacterium which undergoes a transition to swimming for *K* < *K*_*c*_. The dashed line shows the critical spring constant predicted theoretically, *K*_*c*_ ≈ 0.53.

This sharp transition does not originate from a buckling instability of the hook which is only modelled here at the level of a torsional spring^16^. Instead, the instability arises from the two-way coupling between the conformation of the flagella and cell locomotion. To unravel the physics of this instability, we consider in more detail the case of a spherical cell body and two flagella, which is the minimum configuration able to show the instability while capturing the same physics as geometrically-complex cases (see associated movies in supplementary information). The steady-state computational results in this case are shown in the main part of Fig. 4 (symbols and thin lines) for the angle *θ* between the axis of the flagellar filaments and the cell body (A) and for the net lab-frame swimming speed *U* of the cell (B). While the flagella conformation of puller bacteria is independent of the hook stiffness and leads to zero swimming (light red circles), pusher cells clearly display a sudden jump to a wrapped conformation and a net locomotion for a hook stiffness below *K*_*c*_ ≈ 0.79 (dark blue circles).

### Analytical model of the elastohydrodynamic instability

The observed dynamics can be captured by an analytical model demonstrating that swimming occurs as the result of a linear elastohydrodynamic instability. Consider the simple geometrical model illustrated in Fig. 2. Two straight active filaments of length $\ell$ are symmetrically attached on either side of a spherical cell body of radius *a* and are tilted by symmetric angles ±*θ* away from the body surface normal, $\hat{{\bf{N}}}$ (note that the asymmetric tilt mode, not considered here, would lead to a transition in rotation instead of translation). Each filament, elastically attached to the cell body via a hook modelled as a torsion spring of stiffness *K*, pushes on the cell along their tangential direction with propulsive force density $f\hat{{\bf{t}}}$ which results in the swimming of the bacterium with velocity $U\hat{{\bf{y}}}$ (see Fig. 2 for all notation). For CCW motion, the propulsion forces point towards the cell body (*f* < 0) and the cell is a pusher. In contrast, for CW motion, the propulsive forces point away from the cell (*f* > 0) and the swimmer is a puller.

The swimming speed (*U*) and the rate of change of the conformation of the filaments ($\dot{\theta }$) may be obtained by enforcing force and moment balance. Using *c*_||_ and *c*_⊥_ to denote the drag coefficients for a slender filament moving parallel and perpendicular to its tangent respectively (see supplementary material), the balance of forces on the whole cell in the direction of swimming, $\hat{{\bf{y}}}$, is written as

1 $$-\,6\pi \mu aU-2\ell U({c}_{\parallel }{\sin }^{2}\theta +{c}_{\perp }{\cos }^{2}\theta )+\dot{\theta }{\ell }^{2}{c}_{\perp }\,\cos \,\theta =2f\ell \,\sin \,\theta ,$$

where the first two terms are due to the drag on the cell body and on the active filaments due to swimming, the third term is the drag on the filaments due to rotation and the last term is the total propulsive force acting on the cell.

The second equation comes from the balance of moment on each active filament, written in the $\hat{{\bf{z}}}=\hat{{\bf{x}}}\times \hat{{\bf{y}}}$ direction at the attachment point on the cell surface as

2 $$-\frac{{\ell }^{3}}{3}{c}_{\perp }\dot{\theta }+\frac{{\ell }^{2}}{2}U{c}_{\perp }\,\cos \,\theta -K\theta =0,$$

where the first term is the hydrodynamic moment due to rotation of the filament, the second is the hydrodynamic moment due to the swimming drag and the last term is the elastic restoring moment from the hook acting to return the filament to its straight configuration. Combining Eqs (1) and (2) leads to an evolution equation for *θ*

3 $$(\frac{{\ell }^{3}}{3}{c}_{\perp }-\frac{{c}_{\perp }^{2}{\cos }^{2}\theta {\ell }^{4}}{12\pi \mu a+4\ell ({c}_{\parallel }{\sin }^{2}\theta +{c}_{\perp }{\cos }^{2}\theta )})\dot{\theta }=\frac{-f{\ell }^{3}\,\sin \,\theta \,\cos \,\theta {c}_{\perp }}{6\pi \mu a+2\ell ({c}_{\parallel }{\sin }^{2}\theta +{c}_{\perp }{\cos }^{2}\theta )}-K\theta .$$

When the elastic moment dominates, the straight configuration *θ* = 0 is the only steady state, associated with no swimming. If instead the elastic moment is negligible, the swimming states with *θ* = ±π/2 become possible equilibria.

To examine how a variation of the hook stiffness allows transition from one state to the next, we solve Eq. (3) numerically with the appropriate flagellar filament values for a wild type swimming *E. coli* cell and using the magnitude of *f* leading to agreement with the full computations at zero hook stiffness (see supplementary material). We start with small perturbations around *θ* = 0 and compute the long-time steady state of Eq. (3), with results illustrated in Fig. 4 for both pusher (dark red line) and puller (green line). Puller cells never swim for any value of the hook stiffness, and the straight configuration *θ* = 0 is always stable. In contrast, pushers cannot swim for hooks stiffer than a critical value but undergo a sudden transition to direct swimming for softer hooks, in excellent agreement with the computations of the full two-flagella case (symbols in Fig. 4).

The sudden transition to swimming for a critical hook stiffness can be predicted analytically by linearising Eq. (3) near the equilibrium at *θ* = 0, leading to

4 $$(\frac{4\pi \mu a{c}_{\perp }{\ell }^{3}+\frac{1}{3}{c}_{\perp }^{2}{\ell }^{4}}{12\pi \mu a+4{c}_{\perp }\ell })\dot{\theta }\approx -(K+\frac{f{c}_{\perp }{\ell }^{3}}{6\pi \mu a+2{c}_{\perp }\ell })\theta .$$

If *f* is positive (puller) then the configuration with *θ* = 0, which is associated with no swimming *U* = 0, is always linearly stable to small perturbations for any value of *K*. In contrast, pushers with *f* < 0 are linearly unstable for *K* < *K*_*c*_ such that the right-hand side of Eq. (4) becomes positive, i.e. ${K}_{c}=-\,f{c}_{\perp }{\ell }^{3}/(2{c}_{\perp }\ell +6\pi \mu a)$. A linear elastohydrodynamic instability enables therefore pusher bacteria with sufficiently-flexible hooks to dynamically transition to an asymmetric conformation (*θ* ≠ 0) with net swimming (*U* ≠ 0). Note that the simple theoretical model (linear stability and numerical solution of Eq. 3) predicts a critical dimensionless stiffness of *K*_*c*_ ≈ 0.53, in agreement with the computations for the full bacterium model, *K*_*c*_ ≈ 0.79.

## Discussion

How does this swimming instability affect real bacteria? We first note that for the instability to be relevant, the rotary motors need to be spatially distributed around the organism and therefore the instability would not occur if the rotary motors were all located around the same position on the cell body. Lophotrichous bacteria whose multiple flagella are positioned at the pole of the cell (for example, *Helicobacter pylori*) would therefore not be subject to this instability, but peritrichous bacteria such as *E. coli* and *Salmonella enterica* would.

By examining past measurements on the bending stiffness of peritrichous bacterial hooks, we next discover that swimming bacteria are safely on the unstable side, explaining their ability to swim despite the presence of spatially distributed motors. The strength of the torsion spring in our model is given by $K=EI/{\ell }_{h}$, where *EI* is the bending rigidity of the hook and ${\ell }_{h}$ its length. A recent study measured the hook flexibility for different species of peritrichous bacteria by extracting and staining the flagellar hooks and using electron microscopy to observe their deformations due to thermal fluctuations^26^. In this study they found that *E. coli* and *Salmonella enterica* had similar hook bending stiffness in the range $EI\approx 1.6\mbox{--}4.8\times {10}^{-28}\,{{\rm{Nm}}}^{2}$ while singly flagellated bacteria can have stiffer hooks^16^. Re-dimensionalising our computational results above using the viscosity of water (1 mPas) and the pitch (2.22 *μ*m) and frequency (110 Hz) of *E. coli* flagella^7^, we obtain *K*_*c*_ ≈ 9.6 × 10^−19^ Nm. Using a hook length ${\ell }_{h}\approx 55$ nm^2^, we therefore predict a critical hook stiffness of $EI\approx 5.5\times {10}^{-26}\,{{\rm{Nm}}}^{2}$. The hook stiffness of peritrichous bacteria is thus two orders of magnitude smaller than the critical value for the instability. In addition, we predict from the theory (resp. the computations) a factor of approximately 10 (resp. 2.5) between the critical stiffness for the instability and the approximate stiffness at which the bacterium swims with maximum speed, decreasing therefore the gap between the hook stiffness of real bacteria and the critical value allowing to take full advantage of the instability for locomotion.

In summary, we showed theoretically and computationally that pusher peritrichous bacteria can swim by exploiting an elastohydrodynamic instability while pullers never can. This instability is due to the bending rigidity of the hooks and is different from the buckling instability displayed by polar bacteria^16^. The physics of this instability lies in the feedback between the conformation of the flagella and the swimming of the cell. Flagellar filaments create propulsive forces which propel the cell forward. In the frame of the moving cell, the filaments experience hydrodynamic moments aligning them with the direction of swimming. The rigidity of the hook balances these hydrodynamic moments and below a critical rigidity, an elastohydrodynamic instability transitions the filaments from a splayed state to a conformation where they are gathered behind the cell. Our results rationalise the ability of real peritrichous bacteria to swim by showing that they are designed to undergo a successful transition to swimming after each tumble.

## Methods

We give here a brief outline of the computational model, with all details found in supplementary information. Our computational model solves for the instantaneous positions of the flagellar filaments and for the swimming velocity, **U**_*b*_, and angular velocity, **Ω**_*b*_, of the cell body by enforcing mechanical equilibrium at all time (inertia is irrelevant at the scale of bacteria). At low Reynolds numbers, the balance of hydrodynamic forces and moments for the whole cell at its centre leads to a linear relationship between the swimming kinematics of the cell and the angular velocities of each filament, denoted by ***ω***_*i*_ for *i*^th^ filament, of the form

5 $$(\begin{array}{l}{{\bf{U}}}_{b}\\ {{\boldsymbol{\Omega }}}_{b}\end{array})=\sum _{i=1}^{{N}_{f}}\,{{\boldsymbol{\Upsilon }}}_{i}\cdot {{\boldsymbol{\omega }}}_{i},$$

where the tensors **ϒ**_*i*_ depend on the hydrodynamic resistance of each individual component of the cell and on their geometrical arrangements.

The rotation rate of each filament has a prescribed value along its helical axis and we need two additional equations to solve for the other two components. This is obtained by examining the local balance of moments. In the frame of a filament, the swimming velocity and rotations are experienced as background flows which act to tilt the flagellum away from the normal to the motor while the hook applies an elastic restoring moment. The hydrodynamic moment acting on filament *i* may be written as ${{\bf{L}}}_{i}={{\boldsymbol{\Gamma }}}_{i}\cdot {{\bf{U}}}_{b}+{{\boldsymbol{\Lambda }}}_{i}\cdot {{\boldsymbol{\Omega }}}_{b}+{{\boldsymbol{\Delta }}}_{i}\cdot {{\boldsymbol{\omega }}}_{i},$ where the tensors **Γ**_*i*_, **Λ**_*i*_ and **Δ**_*i*_ dependent on the geometry and relative configuration of the flagellar filament and cell body, and are proportional to the fluid viscosity. If we use the unit vector ${\hat{{\bf{N}}}}_{i}$ to denote the direction normal to the cell body surface at the location of the motor and if ${\hat{{\bf{k}}}}_{i}$ is the unit vector along the axis of the filament (see Fig. 2A) then the restoring elastic moment acts along the unit vector ${\hat{{\bf{H}}}}_{i}={\hat{{\bf{k}}}}_{i}\times {\hat{{\bf{N}}}}_{i}$ and the balance between the hydrodynamic moment and restoring elastic moment from the hook is written for all times as

6 $${{\bf{L}}}_{i}\cdot \hat{{{\bf{H}}}_{{\bf{i}}}}+K{\theta }_{i}=0.$$

Finally, we assume that there is no elastic resistance for the filament to move in the direction ${\hat{{\bf{J}}}}_{i}={\hat{{\bf{k}}}}_{i}\times {\hat{{\bf{H}}}}_{i}$ perpendicular to both **k**_*i*_ and ${\hat{{\bf{H}}}}_{i}$ and thus the final moment equation is ${{\bf{L}}}_{i}\cdot \hat{{{\bf{J}}}_{i}}=0$.

## Electronic supplementary material

Figure 3 - Puller flexible swimmer

Figure 3 - Puller stiff swimmer

Figure 3 - Pusher flexible swimmer

Figure 3 - Pusher stiff swimmer

Figure 4 - Flexible spherical swimmer

Figure 4 - Stiff spherical swimmer

Supplementary Information
