# Supplementary material for: Swimming of peritrichous bacteria is enabled by an elastohydrodynamic instability
Source: Sci Rep. 2018 Jul 16;8:10728. doi: 10.1038/s41598-018-28319-8 (PMC6048115; doi:10.1038/s41598-018-28319-8)
Supplement: Supplementary file 7 — Supplementary Information [file 41598_2018_28319_MOESM7_ESM.pdf]

Supplementary Information for: “Swimming of peritrichous bacteria is enabled by an elastohydrodynamic instability”

Emily E. Riley, Debasish Das and Eric Lauga

The supplementary information describes the computational model of a peritrichous bacterium in detail. The schematic diagram of the model is shown in Fig. 2A of the main article. It consists of N_f flagella attached to a spheroidal cell body whose long axis is orientated in the $\hat{\mathbf{z}}$ direction. We first derive the equations relevant for a bacterium with a single flagellum in Supplementary Notes 1, 2 and 3 and then extend them to a bacterium with multiple flagella in Supplementary Note 4. The full numerical algorithm for a peritrichous bacterium is detailed in Supplementary Note 5 while the final Supplementary Note 6 gives additional details about the theoretical model.

Supplementary Note 1: Reference frames

To describe the dynamics of the model bacterium, vectors need to be converted between four different frames namely, the lab ($\hat{\mathbf{X}}-\hat{\mathbf{Y}}-\hat{\mathbf{Z}}$), cell body ($\hat{\mathbf{x}}-\hat{\mathbf{y}}-\hat{\mathbf{z}}$), cell surface ($\hat{\mathbf{T}}^{(1)}-\hat{\mathbf{T}}^{(2)}-\hat{\mathbf{N}}$) and flagellar filament ($\hat{\mathbf{i}}-\hat{\mathbf{j}}-\hat{\mathbf{k}}$) frames as shown in Supplementary Fig. 1 [1].

Lab to cell body frame. To compute the trajectory of the bacterium in the lab frame, we need to express the translational velocity of the bacterium, denoted as $\mathbf{U}_{lab}$, in terms of the translational and angular velocity in the cell body frame, denoted as $\mathbf{U}_b$ and $\mathbf{\Omega}_b$, respectively,

$$\mathbf{U}_{lab} = \mathbf{U}_b \cdot \mathbf{B}_b, \tag{1}$$

where the individual columns of the rotation matrix $\mathbf{B}_b$ consists of the vectors $\mathbf{x}$, $\mathbf{y}$ and $\mathbf{z}$. As the bacterium rotates, the cell body frame rotates with the angular velocity $\mathbf{\Omega}_b$,

$$\frac{d\mathbf{x}}{dt} = \mathbf{\Omega}_b \times \mathbf{x}, \qquad \frac{d\mathbf{y}}{dt} = \mathbf{\Omega}_b \times \mathbf{y}. \tag{2}$$

The unit vectors in the cell body frame $\hat{\mathbf{x}}$ and $\hat{\mathbf{y}}$ are numerically advanced in time, normalised to make them unit magnitude and their cross product is computed to find the unit vector in the z-direction,

$$\hat{\mathbf{x}} = \frac{\mathbf{x}}{|\mathbf{x}|}, \qquad \hat{\mathbf{y}} = \frac{\mathbf{y} - \mathbf{y}\cdot\mathbf{x}}{|\mathbf{y}|}, \qquad \hat{\mathbf{z}} = \hat{\mathbf{x}} \times \hat{\mathbf{y}}. \tag{3}$$

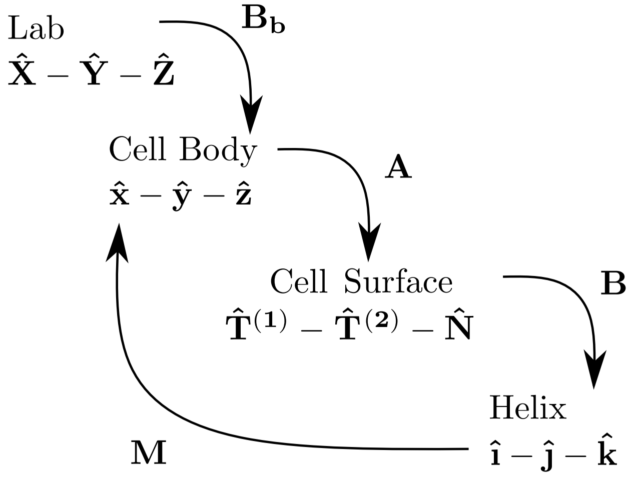

Supplementary Figure 1: Reference Frames. Rotation matrices required to convert vectors between the four reference frames.

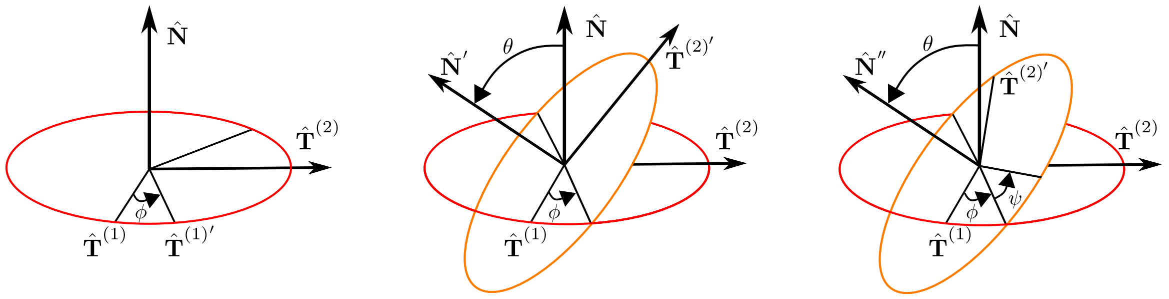

Supplementary Figure 2: Euler Angles. The rotation matrix from the helix frame to the cell surface frame is described using three successive rotations matrices $\mathbf{E}$, $\mathbf{D}$ and $\mathbf{C}$.

Cell body to cell surface frame. We model the cell body as a prolate spheroid with z-direction as the symmetry axis,

$$\frac{x^2 + y^2}{b^2} + \frac{z^2}{c^2} = 1, \tag{4}$$

where b and c are the minor and major semi-axes length respectively ($b < c$), and use the parametric representation,

$$x = b \sin v \cos u, \quad y = b \sin v \sin u, \quad z = c \cos v, \tag{5}$$

where $u = [0, 2\pi)$, $v = [0, \pi]$. The rotation matrix required to convert a vector from the cell body to the cell surface frame is,

$$\mathbf{A} = \begin{pmatrix} \hat{\mathbf{T}}^{(1)} \\ \hat{\mathbf{T}}^{(2)} \\ \hat{\mathbf{N}} \end{pmatrix} = \frac{1}{d} \begin{pmatrix} b \cos u \cos v & b \sin u \cos v & -c \sin v \\ -d \sin u & d \cos u & 0 \\ c \cos u \sin v & c \sin u \sin v & b \cos v \end{pmatrix}, \tag{6}$$

where $d = \sqrt{b^2 \cos^2 v + c^2 \sin^2 v}$ and $\hat{\mathbf{T}}^{(1)}$, $\hat{\mathbf{T}}^{(2)}$ and $\hat{\mathbf{N}}$ are the two unit tangents and normal vector to the surface, respectively.

Cell surface to helix frame. The rotation matrix required to rotate a vector from the flagellum to the cell surface frame, denoted as $\mathbf{B}$, is described with the help of Euler angles and 3 successive rotations as shown in Supplementary Fig. 2. The matrices $\mathbf{E}$, $\mathbf{D}$ and $\mathbf{C}$ describe rotation about the $\hat{\mathbf{N}}$ axis by the precession angle ϕ, $\hat{\mathbf{T}}^{(1)'}$ axis by the tilt angle θ and the helix axis $\hat{\mathbf{k}}$ (or the rotated $\hat{\mathbf{N}}''$ axis) by the roll angle ψ respectively. The dashes superscript signify the change in the unit vectors due to these rotations. Formally $\mathbf{E}$, $\mathbf{D}$ and $\mathbf{C}$ are written as,

$$\mathbf{E} = \begin{pmatrix} \cos\phi & \sin\phi & 0 \\ -\sin\phi & \cos\phi & 0 \\ 0 & 0 & 1 \end{pmatrix}, \quad \mathbf{D} = \begin{pmatrix} 1 & 0 & 0 \\ 0 & \cos\theta & \sin\theta \\ 0 & -\sin\theta & \cos\theta \end{pmatrix}, \quad \mathbf{C} = \begin{pmatrix} \cos\psi & \sin\psi & 0 \\ -\sin\psi & \cos\psi & 0 \\ 0 & 0 & 1 \end{pmatrix}. \tag{7}$$

The final Euler matrix that transforms vectors from the cell surface frame to the flagellum frame is $\mathbf{B} = \mathbf{C} \cdot \mathbf{D} \cdot \mathbf{E}$. Hence, the rotation matrix needed to convert a vector from the helix to the cell body frame is $\mathbf{M} = (\mathbf{B} \cdot \mathbf{A})^{-1}$.

This concludes the description of the different frames of reference and the rotation matrices required to convert vectors between them. As a final calculation, a relation between the angular velocity of the helix and the Euler angles is needed. The rotation rate of each of the Euler angles form a non-orthogonal basis $(\dot{\phi}, \dot{\theta}, \dot{\psi})$, that is written in the orthogonal $\hat{\mathbf{i}} - \hat{\mathbf{j}} - \hat{\mathbf{k}}$ helix frame as,

$$\boldsymbol{\omega}'' = \dot{\phi}\hat{\mathbf{N}} + \dot{\theta}\hat{\mathbf{T}}^{(1)'} + \dot{\psi}\hat{\mathbf{k}} = \begin{pmatrix} \dot{\phi} \sin\theta \sin\psi + \dot{\theta} \cos\psi \\ \dot{\phi} \sin\theta \cos\psi - \dot{\theta} \sin\psi \\ \dot{\psi} + \dot{\phi} \cos\theta \end{pmatrix}, \tag{8}$$

where the relations $\hat{\mathbf{N}} = (\sin\theta \sin\psi, \sin\theta \cos\psi, \cos\theta)$, $\hat{\mathbf{T}}^{(1)'} = (\cos\psi, -\sin\psi, 0)$ and $\hat{\mathbf{k}} = (0, 0, 1)$ have been used.

Supplementary Note 2: Kinematic and dynamic condition

The axial component of the angular velocity of the flagellum in the helix frame, $\boldsymbol{\omega}''$, acts as a forcing for the model. Hence, we need to express the cell body velocities and the forces and torques acting on the bacterium in terms of $\boldsymbol{\omega}''$. The kinematic condition stipulates that the flagellum remains attached to the cell body at all

times requiring them to have the same translational velocity. However, the flagellum is able to rotate relatively to the cell body about its point of attachment. Denoting the vector pointing from the cell centroid to the point of attachment as $\mathbf{r}_v = (b \sin v \cos u, b \sin v \sin u, c \cos v)$, we define a matrix $\mathbf{R}_\epsilon$ to represent cross product of $\mathbf{r}_v$ with a vector $\mathbf{p}$, such that $\mathbf{r}_v \times \mathbf{p} = \mathbf{R}_\epsilon \cdot \mathbf{p}$,

$$\mathbf{R}_\epsilon = \begin{pmatrix} 0 & -r_{v,3} & r_{v,2} \\ r_{v,3} & 0 & -r_{v,1} \\ -r_{v,2} & r_{v,1} & 0 \end{pmatrix}. \tag{9}$$

The angular velocity of the flagellum in the cell body frame, applied at the point of attachment $\mathbf{r}_v$, is $\boldsymbol{\omega}' = \mathbf{M}\cdot\boldsymbol{\omega}''$. Using rigid body dynamics equations, $\boldsymbol{\Omega}_f = \boldsymbol{\Omega}_b + \boldsymbol{\omega}'$, $\mathbf{U}_f = \mathbf{U}_b + \mathbf{R}_\epsilon \cdot \boldsymbol{\omega}'$, the kinematic boundary condition can be formally written as,

$$\begin{pmatrix} \mathbf{U}_f \\ \boldsymbol{\Omega}_f \end{pmatrix} = \begin{pmatrix} \mathbf{U}_b \\ \boldsymbol{\Omega}_b \end{pmatrix} + \tilde{\mathbf{M}} \begin{pmatrix} \mathbf{0} \\ \mathbf{M}\cdot\boldsymbol{\omega}'' \end{pmatrix}, \quad \text{where} \quad \tilde{\mathbf{M}} = \begin{pmatrix} \mathbf{I} & \mathbf{R}_\epsilon \\ \mathbf{0} & \mathbf{I} \end{pmatrix}, \tag{10}$$

and $\mathbf{I}$ is a 3×3 identity matrix.

The bacterium as a whole is force-free and torque-free. Denoting the forces acting on the cell body and flagellum as $\mathbf{F}_b$ and $\mathbf{F}_f$ and torques as $\mathbf{L}_b$ and $\mathbf{L}_f$ respectively, the dynamic boundary condition in the cell body frame reads,

$$\begin{pmatrix} \mathbf{F}_b \\ \mathbf{L}_b \end{pmatrix} + \begin{pmatrix} \mathbf{F}_f \\ \mathbf{L}_f \end{pmatrix} = \begin{pmatrix} \mathbf{0} \\ \mathbf{0} \end{pmatrix}. \tag{11}$$

Since the Reynolds number relevant for bacteria locomotion is small, we can apply Stokes drag law for both cell body and flagella,

$$\begin{pmatrix} \mathbf{F}_b \\ \mathbf{L}_b \end{pmatrix} = \mathbf{R}_b \begin{pmatrix} \mathbf{U}_b \\ \boldsymbol{\Omega}_b \end{pmatrix}, \quad \begin{pmatrix} \mathbf{F}_f \\ \mathbf{L}_f \end{pmatrix} = \mathbf{R}_f \begin{pmatrix} \mathbf{U}_f \\ \boldsymbol{\Omega}_f \end{pmatrix}, \tag{12}$$

and rewrite equation (11) as,

$$\mathbf{R}_b \begin{pmatrix} \mathbf{U}_b \\ \boldsymbol{\Omega}_b \end{pmatrix} + \mathbf{R}_f \begin{pmatrix} \mathbf{U}_f \\ \boldsymbol{\Omega}_f \end{pmatrix} = \begin{pmatrix} \mathbf{0} \\ \mathbf{0} \end{pmatrix}, \tag{13}$$

where the expressions for the resistance matrices of the cell body and the flagellum denoted as $\mathbf{R}_b$ and $\mathbf{R}_f$, respectively are computed in the following notes. Finally, substituting for $\mathbf{U}_f$ and $\boldsymbol{\Omega}_f$ (using equation 13) in equation (10), we can express the angular velocities of the body linearly in terms of $\boldsymbol{\omega}''$,

$$\begin{pmatrix} \mathbf{U}_b \\ \boldsymbol{\Omega}_b \end{pmatrix} = -(\mathbf{R}_b + \mathbf{R}_f) \cdot \mathbf{R}_f \cdot \tilde{\mathbf{M}} \begin{pmatrix} \mathbf{0} \\ \mathbf{M}\cdot\boldsymbol{\omega}'' \end{pmatrix} = \begin{pmatrix} \mathbf{P}_1 & \mathbf{P}_2 \\ \mathbf{P}_3 & \mathbf{P}_4 \end{pmatrix} \begin{pmatrix} \mathbf{0} \\ \boldsymbol{\omega}'' \end{pmatrix}, \tag{14}$$

so that $\mathbf{U}_b = \mathbf{P}_2 \cdot \omega''$ and $\boldsymbol{\Omega}_b = \mathbf{P}_4 \cdot \boldsymbol{\omega}''$. The analytical expressions for $\mathbf{P}$ are cumbersome and computed numerically.

Resistance matrix of cell body in cell body frame. For a spheroidal cell body, the 6×6 resistance matrix is [2]

$$\mathbf{R_b} = -\begin{pmatrix} R_b(1,1) & 0 & 0 & 0 & 0 & 0 \\ 0 & R_b(2,2) & 0 & 0 & 0 & 0 \\ 0 & 0 & R_b(3,3) & 0 & 0 & 0 \\ 0 & 0 & 0 & R_b(4,4) & 0 & 0 \\ 0 & 0 & 0 & 0 & R_b(5,5) & 0 \\ 0 & 0 & 0 & 0 & 0 & R_b(6,6) \end{pmatrix}, \tag{15}$$

with diagonal elements given by

$$R_b(1,1) = R_b(2,2) = \frac{6\pi\mu c(16e^3)}{3(2e + (3e^2 - 1)L)}, \tag{16}$$

$$R_b(3,3) = \frac{6\pi\mu c(8e^3)}{3(-2e + (1 + e^2)L)}, \tag{17}$$

$$R_b(4,4) = R_b(5,5) = \frac{8\pi\mu c^3(4e^3(2 - e^2))}{3(-2e + (1 + e^2)L)}, \tag{18}$$

$$R_b(6,6) = \frac{8\pi\mu c^3(4e^3(1 - e^2))}{3(2e - (1 - e^2)L)}, \tag{19}$$

where $L = \ln\{(1 + e)/(1 - e)\}$ and $e = \sqrt{1 - b^2/c^2}$ is the eccentricity of the spheroid.

Resistance matrix of flagellum in cell body frame. The resistance matrix of a helix in an infinite fluid medium denoted as $\mathbf{R}''_f$ can be computed either using resistive force theory [2] or slender body theory [3]. The force and torque balance equations in the flagellum frame (denoted with double dashes superscript) are

$$\begin{pmatrix} \mathbf{F}''_f \\ \mathbf{L}''_f \end{pmatrix} = \mathbf{R}''_f \begin{pmatrix} \mathbf{U}''_f \\ \mathbf{\Omega}''_f \end{pmatrix}, \quad \text{where} \;\; \mathbf{R}''_f = \begin{pmatrix} \mathbf{D_a}'' & \mathbf{D_b}'' \\ \mathbf{D_b}''^T & \mathbf{D_c}'' \end{pmatrix}. \tag{20}$$

In order to find the resistance matrix of the flagellum in the cell body frame, we need to convert equation (20) from the helix to the cell surface and subsequently to cell body frame. Denoting the vectors in the cell surface frame with a single dash and recalling that we need to multiple a vector with $\mathbf{M} = (\mathbf{BA})^{-1}$ to convert it from the helix to the cell surface frame, the force-torque balance equation in the latter frame reads

$$\begin{pmatrix} \mathbf{F}'_f \\ \mathbf{L}'_f \end{pmatrix} = \begin{pmatrix} \mathbf{M} & \mathbf{0} \\ \mathbf{0} & \mathbf{M} \end{pmatrix} \begin{pmatrix} \mathbf{D_a}'' & \mathbf{D_b}'' \\ \mathbf{D_b}''^T & \mathbf{D_c}'' \end{pmatrix} \begin{pmatrix} \mathbf{M}^{-1} & \mathbf{0} \\ \mathbf{0} & \mathbf{M}^{-1} \end{pmatrix} \begin{pmatrix} \mathbf{U}'_f \\ \mathbf{\Omega}'_f \end{pmatrix}. \tag{21}$$

Using rigid body dynamics equations, we have relations between velocities $\mathbf{\Omega} = \mathbf{\Omega}'$, $\mathbf{U} = \mathbf{U}' + \mathbf{R}_\epsilon \cdot \mathbf{\Omega}'$ and forces and torques $\mathbf{F} = \mathbf{F}'$, $\mathbf{L} = \mathbf{L}' + \mathbf{R}_\epsilon \cdot \mathbf{F}'$ in the cell body and cell surface frame (denoted by single dashes), written compactly as,

$$\begin{pmatrix} \mathbf{F}_f \\ \mathbf{L}_f \end{pmatrix} = \begin{pmatrix} \mathbf{I} & \mathbf{0} \\ \mathbf{R}_\epsilon & \mathbf{I} \end{pmatrix} \begin{pmatrix} \mathbf{F}'_f \\ \mathbf{L}'_f \end{pmatrix}, \qquad \begin{pmatrix} \mathbf{U}_f \\ \mathbf{\Omega}_f \end{pmatrix} = \begin{pmatrix} \mathbf{I} & \mathbf{R}_\epsilon \\ \mathbf{0} & \mathbf{I} \end{pmatrix} \begin{pmatrix} \mathbf{U}'_f \\ \mathbf{\Omega}'_f \end{pmatrix}. \tag{22}$$

Combining equations (21) and (22), the desired force and torque balance equation for the flagellum in the cell body frame is,

$$\begin{pmatrix} \mathbf{F}_f \\ \mathbf{L}_f \end{pmatrix} = \mathbf{R}_f \begin{pmatrix} \mathbf{U}_f \\ \mathbf{\Omega}_f \end{pmatrix}, \tag{23}$$

where

$$\mathbf{R}_f = \begin{pmatrix} \mathbf{I} & \mathbf{0} \\ \mathbf{R}_\epsilon & \mathbf{I} \end{pmatrix} \begin{pmatrix} \mathbf{M} & \mathbf{0} \\ \mathbf{0} & \mathbf{M} \end{pmatrix} \begin{pmatrix} \mathbf{D}''_a & \mathbf{D}''_b \\ \mathbf{D}''^T_b & \mathbf{D}''_c \end{pmatrix} \begin{pmatrix} \mathbf{M}^{-1} & \mathbf{0} \\ \mathbf{0} & \mathbf{M}^{-1} \end{pmatrix} \begin{pmatrix} \mathbf{I} & -\mathbf{R}_\epsilon \\ \mathbf{0} & \mathbf{I} \end{pmatrix}. \tag{24}$$

Supplementary Note 3: Torque balance on a single flagellum

Viscous torque. Using equation (23), we can obtain a relation between the force and torque acting on the flagellum in the helix frame and the cell body and flagellum velocities,

$$\begin{pmatrix} \mathbf{F}''_f \\ \mathbf{L}''_f \end{pmatrix} = \begin{pmatrix} \mathbf{D}''_a & \mathbf{D}''_b \\ \mathbf{D}''^T_b & \mathbf{D}''_c \end{pmatrix} \begin{pmatrix} \mathbf{M}^{-1} \cdot (\mathbf{U_b} - \mathbf{R}_\epsilon \mathbf{\Omega_b}) \\ \mathbf{M}^{-1} \cdot \mathbf{\Omega_b} + \boldsymbol{\omega}'' \end{pmatrix}. \tag{25}$$

Substituting for the cell body velocities using equation (14), the torque acting on the flagellum can be linearly expressed in terms of $\boldsymbol{\omega}''$,

$$\mathbf{L}''_f = \left[\mathbf{D}''^T_b \cdot \mathbf{M}^{-1} \cdot \{\mathbf{P}_2 - \mathbf{R}_\epsilon \cdot \mathbf{P}_4\} + \mathbf{D}''_c \cdot \mathbf{M}^{-1} \cdot \mathbf{P}_4 + \mathbf{D}''_c\right] \cdot \boldsymbol{\omega}'' \tag{26}$$

Note that from equation (26), the operators $\mathbf{\Gamma}_i$, $\mathbf{\Lambda}_i$ and $\mathbf{\Delta}_i$ used in the main text may be deduced (for simplicity we have dropped the $''$ and the subscript f in the main text).

Elastic torque. The hook is modelled as an elastic spring of stiffness K and owing to its small size, its hydrodynamics is neglected. The neutral position of the hook is when the helix axis is aligned with the cell surface normal, i.e., $\theta = 0$. We assume that only deviations of the tilt angle θ from the surface normal $\hat{\mathbf{N}}$ generates an elastic torque. The hook is assumed to be stiff to torsion and to act as a perfect torque transmitter from the motor embedded in the cell body to the flagellar filament, valid for steady motor rotation [4]. As a consequence, the elastic torque is only dependent on θ and independent of the precession ϕ and roll angle ψ,

$$\mathbf{L}_e = K\theta\hat{\mathbf{H}}, \quad \text{where} \;\; \hat{\mathbf{H}} = \frac{\hat{\mathbf{k}} \times \hat{\mathbf{N}}_{\hat{\mathbf{i}}\hat{\mathbf{j}}\hat{\mathbf{k}}}}{|\hat{\mathbf{k}} \times \hat{\mathbf{N}}_{\hat{\mathbf{i}}\hat{\mathbf{j}}\hat{\mathbf{k}}}|}, \tag{27}$$

describes the direction in which the torque acts. The helix axis and the normal to the cell surface written in the helix frame are $\hat{\mathbf{k}} = (0, 0, 1)$ and $\hat{\mathbf{N}}_{\hat{\mathbf{i}}\hat{\mathbf{j}}\hat{\mathbf{k}}} = \mathbf{B} \cdot (0, 0, 1)$, respectively that gives $\hat{\mathbf{H}} = (-\cos\psi, \sin\psi, 0)$. The desired expression for the elastic torque in terms of the Euler angles and hook stiffness K is,

$$\mathbf{L}_e = K\theta(-\cos\psi, \sin\psi, 0). \tag{28}$$

As there is no hydrodynamic interaction between the cell body and the flagellum, it is possible for the flagellum to enter the cell body. In order to prevent such overlap, we add a steric repulsive torque between the cell body

and the flagellum by increasing the stiffness of the spring with a smooth step function as θ increases beyond a certain value,

$$f(\theta) = f_{max}\left(1 - \frac{1}{1+(\theta/\theta_0)^{k_s}}\right), \tag{29}$$

where θ_0 is the value at which $f \to f_{max}$ and k_s is the steepness. The value of θ_0 is taken to be $\pi/2$ and f_{max} and k_s are chosen such that the critical value of hook stiffness, K_c, remains unaffected. Increasing the value of f_{max} or k_s has negligible effects on the observed dynamics but imposes a stricter restriction on the time step. The modified expression for the elastic torque is,

$$\mathbf{L}_e = \left(K\theta + 100\left(1 - \frac{1}{1+\left(\frac{2\theta}{\pi}\right)^{70}}\right)\right)\hat{\mathbf{H}}, \tag{30}$$

that prevents the flagellum from entering the body without changing the physics of the elastohydrodynamic instability.

Final torque balance. In order to balance torques on the flagellum with a fixed rotation $\boldsymbol{\omega}''$ along the helix axis, we form an orthonormal basis $\hat{\mathbf{H}} - \hat{\mathbf{k}} - \hat{\mathbf{J}}$, where $\hat{\mathbf{J}} = \hat{\mathbf{H}} \times \hat{\mathbf{k}} = (\sin\psi, \cos\psi, 0)$. The torque along the $\hat{\mathbf{k}}$ axis is fixed as the helix rotates with a constant angular velocity in this direction. The torque balance equations in the $\hat{\mathbf{J}}$ and $\hat{\mathbf{H}}$ direction are,

$$\hat{\mathbf{J}} \cdot \mathbf{L}''_f = 0, \quad \hat{\mathbf{H}} \cdot (\mathbf{L}''_f + \mathbf{L}_e) = 0. \tag{31}$$

Equation (31) gives rise to a linear system of equations that can be solved for $\boldsymbol{\omega}''(1)$ and $\boldsymbol{\omega}''(2)$ for a prescribed dimensional angular velocity, $\boldsymbol{\omega}''(3) = 2\pi$ for a pusher and $\boldsymbol{\omega}''(3) = -2\pi$ for a puller. As a final calculation, the cell body velocities can be computed using equation (14) and the temporal derivatives of the Euler angles can be computed by inverting equation (8)

$$\begin{pmatrix}\dot{\phi}\\ \dot{\theta}\\ \dot{\psi}\end{pmatrix} = \begin{pmatrix}\sin\theta\sin\psi & \cos\psi & 0\\ \sin\theta\cos\psi & -\sin\psi & 0\\ \cos\theta & 0 & 1\end{pmatrix}^{-1}\begin{pmatrix}\boldsymbol{\omega}''(1)\\ \boldsymbol{\omega}''(2)\\ \boldsymbol{\omega}''(3)\end{pmatrix}, \tag{32}$$

from which the Euler angles can be advanced in time using a fourth-order Runge-Kutta scheme.

Supplementary Note 4: Extension to peritrichous bacteria

It is straightforward to extend the monoflagellar bacterium model described above to a multiflagellar one, applicable to *E. coli* using the parameters listed in Supplementary Table 1. Following previous work on bacterium models [7], we use a tapered left-handed helix to represent a flagellum such that its radius at the attachment point is zero,

$$\mathbf{h} = R_h\left(1 - e^{-(k_e s)^2}\right)\sin(2\pi s)\hat{\mathbf{i}} + R_h\left(1 - e^{-(k_e s)^2}\right)\cos(2\pi s)\hat{\mathbf{j}} + s\hat{\mathbf{k}}, \quad s = [0, L_\lambda], \tag{33}$$

Parameters	Symbols	Values	Dimensionless values
Cell Body (Major Axis Radius)	c	1.25 µm	0.56
Cell Body (Minor Axis Radius)	b	0.44 µm	0.2
Number of Flagella	N_f	1–7 [5]	-
Helix Rotation Frequency	ν	110 Hz	1
Helix Radius	R_h	0.2 µm	0.09
Helix Wavelength	λ	2.22 µm	1
Helix Pitch Angle	$\beta = \tan^{-1}(2\pi R_h/\lambda)$	30°	-
Helix Contour Length	L_c	7.1 µm	3.20
Helix Axis Length	L_λ	6.18 µm	2.78
Helix Filament Radius	r_f	0.012 µm	0.0054
Bacteria Hook Length	l_h	0.055 µm	0.025
Number of Turns	$n = L_\lambda/\lambda$	2.8	-

Supplementary Table 1: Typical wild type *E. coli* parameters obtained from reference [6] and used in this study.

where $k_e = 3\pi/2$. The resistance matrix of the helix computed using slender body theory [3] with the dimensionless parameters from Supplementary Table 1 is,

$$\mathbf{R_f}'' = \begin{pmatrix} -4.9843 & -0.0278 & -0.0277 & 0.0224 & -10.9573 & -0.0318 \\ -0.0278 & -5.8073 & 0.0244 & 11.4012 & 0.0188 & -0.0388 \\ -0.0277 & 0.0244 & -3.6158 & 0.0026 & -0.1732 & -0.0855 \\ 0.0224 & 11.4012 & 0.0026 & -27.1636 & 0.0500 & -0.0020 \\ -10.9573 & 0.0188 & -0.1732 & 0.0500 & -26.9677 & -0.1007 \\ -0.0318 & -0.0388 & -0.0855 & -0.0020 & -0.1007 & -0.0633 \end{pmatrix}. \quad (34)$$

We revisit the kinematic relationship,

$$\sum_{i=1}^{N_f} \begin{pmatrix} \mathbf{U}_{f,i} \\ \boldsymbol{\Omega}_{f,i} \end{pmatrix} = \begin{pmatrix} \mathbf{U}_b \\ \boldsymbol{\Omega}_b \end{pmatrix} + \sum_{i=1}^{N_f} \left[\tilde{\mathbf{M}}_i \begin{pmatrix} \mathbf{0} \\ \mathbf{M}_i \cdot \boldsymbol{\omega}'_i \end{pmatrix} \right], \quad (35)$$

and dynamic conditions for the bacterium with N_f flagella attached on its surface

$$\sum_{i=1}^{N_f} \begin{pmatrix} \mathbf{F}_{f,i} \\ \mathbf{L}_{f,i} \end{pmatrix} + \begin{pmatrix} \mathbf{F}_b \\ \mathbf{L}_b \end{pmatrix} = 0, \quad (36)$$

where summation is used to imply adding the contributions of each individual flagellum. Similar to the single flagellum case in Supplementary Note 2, using equations (35) and (36), we can substitute for $\mathbf{U}_{f,i}$ and $\boldsymbol{\Omega}_{f,i}$ to obtain a linear relation between the cell body velocities and the angular velocities of all the flagella $\boldsymbol{\omega}''_i$,

$$\begin{pmatrix} \mathbf{U}_b \\ \boldsymbol{\Omega}_b \end{pmatrix} = -\left(\mathbf{R}_b + \sum_{i=1}^{N_f} \mathbf{R}_{f,i} \right)^{-1} \left[\sum_{i=1}^{N_f} \mathbf{R}_{f,i} \cdot \tilde{\mathbf{M}}_{\mathbf{i}} \begin{pmatrix} \mathbf{0} \\ \mathbf{M}_i \cdot \boldsymbol{\omega}''_i \end{pmatrix} \right] = \sum_{i=1}^{N_f} \begin{pmatrix} \mathbf{P}_1^i & \mathbf{P}_2^i \\ \mathbf{P}_3^i & \mathbf{P}_4^i \end{pmatrix} \begin{pmatrix} \mathbf{0} \\ \boldsymbol{\omega}''_i \end{pmatrix}, \quad (37)$$

so that $\mathbf{U}_b = \sum_{i=1}^{N_f} \mathbf{P}_2^i \cdot \boldsymbol{\omega}''_i$ and $\boldsymbol{\Omega}_b = \sum_{i=1}^{N_f} \mathbf{P}_4^i \cdot \boldsymbol{\omega}''_i$. Note that from equation (37), we can read off what the operators $\boldsymbol{\Upsilon}_i$ are for each flagellum in Eq. 1 of the main text.

Following a similar procedure as in Supplementary Note 3, we can express the viscous torque acting on each flagellum j in terms of $\boldsymbol{\omega}''_i$,

$$\mathbf{L}''_{f,j} = \sum_{i=1}^{N_f} \left[\mathbf{D}''^T_b \cdot \mathbf{M}^{-1} \cdot \{ \mathbf{P}_2 - \mathbf{R}_\epsilon \cdot \mathbf{P}_4 \} + \mathbf{D}''_c \cdot \mathbf{M}^{-1} \cdot \mathbf{P}_4 + \mathbf{D}''_c \right]_{ji} \cdot \boldsymbol{\omega}''_i, \quad j = 1, \ldots, N_f \quad (38)$$

Finally, torque balance equation (31) is applied to each flagellum j,

$$\hat{\mathbf{J}}_j \cdot \mathbf{L}''_{f,j} = 0, \quad \hat{\mathbf{H}}_j \cdot (\mathbf{L}''_{f,j} + \mathbf{L}_e) = 0, \quad j = 1, \ldots, N_f \quad (39)$$

giving rise to $2N_f$ linear equations for the $2N_f$ unknowns $\boldsymbol{\omega}''_j(1)$ and $\boldsymbol{\omega}''_j(2)$ while $\boldsymbol{\omega}''_j(3) = \pm 2\pi$ is prescribed for each flagellum. Having computed the angular velocities of each flagellum, we can evaluate the cell body velocities and the temporal derivatives of the Euler angles of each flagellum j using equation (37) and (32), respectively.

Supplementary Note 5: Numerical implementation

1. The geometry of the model bacterium is specified in terms of dimensionless parameters as shown in Supplementary Table 1 and the elastic spring stiffness K.

2. The resistance of the cell body, $\mathbf{R}_b$, and the flagella, $\mathbf{R}_f$, are calculated in the cell body frame using slender body theory.

3. The point of attachment on the cell surface for each flagellum is specified and the corresponding matrices $\mathbf{A}^i$ are calculated.

4. The initial Euler angles for each flagellum at time $t = 0$ are specified, ϕ_i, ψ_i and $\theta_i \neq 0$, so as to avoid singularity when using equation (32). In practice, θ_i never attains a value of zero exactly in our simulations but may get close to it.

5. The matrix $\mathbf{M}^i$ required to convert a vector from helix frame to cell surface frame is computed and the matrices $\mathbf{P}_2^i$ and $\mathbf{P}_4^i$ are computed.

6. The linear system of equations (39) is solved to obtain the angular velocities of the flagella $\boldsymbol{\omega}_i''(1)$ and $\boldsymbol{\omega}_i''(2)$ by prescribing $\boldsymbol{\omega}_i''(3) = \pm 2\pi$.

7. The body velocities $\mathbf{U}_b$ and $\boldsymbol{\Omega}_b$ and the lab velocity $\mathbf{U}_{lab}$ are computed. The position of the bacterium is then advanced as $\dot{\mathbf{X}} = \mathbf{U}_{lab}$ and its trajectory is recorded in time.

8. The temporal derivatives of the Euler angles are computed and advanced in time using the fourth order Runge-Kutta scheme.

9. The body velocities and Euler angles have small periodic oscillations due to the helical geometry of the flagella [8]. We use the filter command in MATLAB [9] to compute the average values of these quantities. Steps 5–8 are repeated with the new Euler angles obtained for each flagellum until a steady state is reached.

Supplementary Note 6: Theoretical Model

As detailed in the main article, the straight filament model is able to capture the elastohydrodynamic instability and provides an analytical expression for the critical hook stiffness $K_c = -fc_\perp \ell^3/(2c_\perp \ell + 6\pi\mu a)$ (note that $a = 0.5\,\mu\text{m}$ is the radius of the sphere used in theoretical and computational model in Fig. 4 of the main text). In order to allow comparison between the active filament and helical model, we use the maximum swimming speed $U_{max} = 0.0815$ that occurs at $\theta_{max} = 1.4686$ from the computational model to compute the corresponding constant active force strength, $f = -0.1425$, using the force balance Eq. (1) in the main text of the paper with $\dot{\theta} = 0$. The filament is modelled as a slender prolate spheroid having minor and major axes length as $2r_f$ and l, respectively and its resistance coefficients are [10],

$$c_\parallel = \frac{2\pi\mu}{\log(l/r_f) - 1/2}, \tag{40}$$

$$c_\perp = \frac{4\pi\mu}{\log(l/r_f) + 1/2}. \tag{41}$$

Using the required values from Supplementary Table 1 and non-dimensionalising lengths with λ, so that $l = L_c = 3.20$, $r_f = 0.0054$ and $a = 0.225$, these drag coefficients are found to be $c_\parallel = 1.068$ and $c_\perp = 1.826$ which gives the critical hook stiffness $K_c = 0.53$ reported in the main text of the paper.

Supplementary References

[1] Marcos. *Bacteria in shear flow.* PhD thesis, Dept. of Mechanical Engineering, MIT, 2011.

[2] S. Kim and S. J. Karrila. *Microhydrodynamics: Principles and Selected Applications.* Dover Publications, Inc., Minieola, New York, 2005.

[3] R. E. Johnson. An improved slender-body theory for Stokes flow. *J. Fluid Mech.*, 99:411–431, 1980.

[4] Y. Sowa and R. M. Berry. Bacterial flagellar motor. *Q. Rev. Biophys.*, 41:103–132, 2008.

[5] G. N. Cohen-Ben-Lulu, N. R. Francis, E. Shimoni, D. Noy, Y. Davidov, K. Prasad, Y. Sagi, G. Cecchini, R. M. Johnstone, and M. Eisenbach. The bacterial flagellar switch complex is getting more complex. *EMBO J.*, 27:1134–44, 2008.

[6] N. C. Darnton, L. Turner, S. Rojevsky, and H. C. Berg. On torque and tumbling in swimming *Escherichia coli*. *J. Bacteriol.*, 189:1756–1764, 2007.

[7] J. J. L. Higdon. The hydrodynamics of flagellar propulsion: helical waves. *J. Fluid Mech.*, 94:331–351, 1979.

[8] Y. Hyon, T. R. Powers, R. Stocker, and H. C. Fu. The wiggling trajectories of bacteria. *J. Fluid Mech.*, 705:58–76, 2012.

[9] MATLAB R2015B. The Mathworks, Inc. *Natick, MA*, 2015.

[10] A. T. Chwang and T. Y. Wu. Hydromechanics of low-Reynolds-number flow. Part 2. Singularity method for Stokes flows. *J. Fluid Mech.*, 67:787–815, 1975.
